# Assessment of Prescribing and Monitoring Habits for Patients Taking an Antiarrhythmic and Concomitant QTc-Prolonging Antibiotic

**DOI:** 10.3390/pharmacy5040061

**Published:** 2017-11-01

**Authors:** Kelsey Noss, Sandra M. Aguero, Travis Reinaker

**Affiliations:** Einstein Medical Center-Philadelphia, 5501 Old York Road, Philadelphia, PA 19141, USA; kelsey.noss12@gmail.com (K.N.); aguersan@einstein.edu (S.M.A.)

**Keywords:** QTc-prolongation, electrocardiogram, antiarrhythmic, macrolide, fluoroquinolone, drug-interaction

## Abstract

Patients may intermittently require antimicrobial therapy with a QTc-prolonging antibiotic, which presents a challenge for prescribers of patients already taking a QTc-prolonging antiarrhythmic. Manufacturers recommend close monitoring for evidence of QTc-prolongation with the concomitant use of QTc-prolonging medications, but the monitoring parameters are not well-defined. Previous studies recommend a surveillance electrocardiogram (EKG) be completed both before and after the initiation of QTc-prolonging medications, but it is unknown to what degree EKGs displaying the QTc-interval are used to alter physician order entry and pharmacist order verification during concomitant therapy. A retrospective chart review was conducted between October 2015–September 2016 to assess prescribing and monitoring habits for patients taking an antiarrhythmic and a concomitant QTc-prolonging antibiotic. Of the 42 patients who received at least one dose of two QTc-prolonging agents, 36 (85.7%) received a baseline EKG, and 23 (63.8%) received a follow-up EKG. Pharmacists intervened on this drug–drug interaction and recommended follow-up EKGs only three times (8.3%) and offered alternative therapy recommendations once (2.8%). The QTc-interval was not optimally monitored in some instances for patients concomitantly receiving two QTc-prolonging agents. These results stress the importance of inter-professional communication to place an emphasis on follow-up monitoring or use of alternative therapy agents to avoid the drug–drug interaction altogether.

## 1. Introduction

The QT-interval on an electrocardiogram (EKG) represents the depolarization and repolarization of cardiac ventricles. On a 12-lead EKG, the QT-interval is measured from the beginning of the QRS complex to the end of the T wave as it returns to baseline. Several factors such as gender, heart rate, underlying arrhythmias, and conduction defects influence the QT-interval. The QT-interval will vary depending on heart rate. To standardize the measurement to a heart rate of 60 beats per minute, the QT-interval is corrected and referred to as the QTc [1]. The QTc allows for comparison of the QT-interval across a range of heart rates. The most universally adopted method for correcting QT-intervals for heart rate is the Bazett’s formula (Corrected QT-interval (QTc): QT/√RR in seconds; RR is the interval from the peak of one QRS complex to the peak of the next as shown on an electrocardiogram) [1]. QTc-prolongation is defined as a QTc-interval of >450 milliseconds (ms) in males, and >470 ms in females, and can predispose patients to life-threatening ventricular arrhythmias. Several medications have been implicated in the prolongation of the QTc-interval. A complete resource of medications, stratified according to QTc-prolonging risk, can be found at crediblemeds.com. QTc-prolongation can occur in up to 10% of patients taking QTc-prolonging antiarrhythmics (including amiodarone), and <1% of patients taking macrolide or fluoroquinolone antibiotics. The concomitant use of two QTc-prolonging medications increases this risk [1]. Manufacturers recommend close monitoring for evidence of QTc-prolongation with the concomitant use of QTc-prolonging medications, while previous studies recommend that offending drugs should be discontinued in patients who develop a prolonged QTc-interval >500 ms, or an increase in QTc-interval of >60 ms on follow-up EKG [2,3,4,5,6,7,8]. However, it is unknown whether EKGs are used to alter prescribing and monitoring habits when these medications are combined. This is the first study to observe the real-life prescribing and monitoring habits for patients taking an antiarrhythmic and a concomitant QTc-prolonging antibiotic at a large, academic medical center.

## 2. Materials and Methods

A retrospective chart review, exempt from IRB-approval, was completed to observe the prescribing and monitoring habits for patients taking an antiarrhythmic and a concomitant QTc-prolonging antibiotic. This study included patients admitted to Einstein Medical Center-Philadelphia from 1 October 2015 to 30 September 2016. Patients were identified with an electronic report of drug interaction alerts that had advised pharmacists during order verification of the increased risk of a QTc-prolonging effect between two medications. Patients taking amiodarone upon admission who also received at least one concomitant dose of ciprofloxacin, moxifloxacin, or azithromycin during admission were included. Gender, QTc-prolonging medication, medication dose, pharmacist interventions, presence or absence of a baseline (while on amiodarone only) and follow-up (while on amiodarone and a QTc-prolonging antibiotic), QTc-interval, change in QTc-interval, and therapy modification and justification were collected. The formula used to calculate the corrected QT-interval was Bazett’s formula. Descriptive statistics, including the median and range, were used to analyze patient demographics, prescriptions, and monitoring data.

## 3. Results

A total of 78 patients were assessed, and 42 patients received concomitant QTc-prolonging agents. The most commonly prescribed medication regimen was azithromycin added to home amiodarone therapy in 23 patients (54.5%). Thirty-six out of 42 patients (85.7%) previously taking amiodarone received a baseline EKG (Table 1). The median male QTc-interval was 473 ms (range: 405–602 ms), and the median female QTc-interval was 470 ms (range: 435–599 ms) (Table 1). Of the male patients who received a baseline EKG, nine out of 14 (64.2%) had a prolonged QTc-interval (>450 ms). Of the female patients who received a baseline EKG, 12 out of 22 (54.5%) had a prolonged QTc-interval (>470 ms) (Table 1). Of the 36 patients who received a baseline EKG, a pharmacist recommended a follow-up EKG on three occasions (8.3%) (Table 1). Twenty-three out of 36 patients (63.8%) received a follow-up EKG. The median male QTc-interval was 481 ms (range: 440–628 ms), and the median female QTc-interval was 484 ms (range: 384–645 ms) (Table 1). Of the male patients who received a follow-up EKG, two out of eight (25%) had a prolonged QTc-interval (>450 ms). Of the female patients who received a follow-up EKG, 11 out of 15 (73.3%) had a prolonged QTc-interval (>470 ms) (Table 1). Ten out of 23 patients (43.5%) had a QTc-interval >500 ms or an increase in QTc-interval of >60ms on follow-up EKG, but in only three out of 23 instances (13%), therapy was either discontinued or a different antimicrobial was utilized (Table 1). Of these three patients, two (66.7%) experienced a QTc-interval increase to >600 ms without arrhythmia, and one (33.3%) developed torsades de pointes before alternative therapy was utilized. (Table 1).

## 4. Discussion

Manufacturers recommend close monitoring for evidence of QTc-prolongation with the concomitant use of QTc-prolonging medications, but the monitoring parameters are not well-defined [2,3,4,5]. Previous studies have recommended that surveillance EKGs be completed before and after initiation of QTc-prolonging medications [1,6,7,8]. This observational study demonstrated that despite prescribers ordering baseline EKGs on most patients, 36.2% of patients still did not receive a follow-up EKG. In the presence of a follow-up EKG, “The Significance of QT-Interval in Drug Development” published in the British Journal of Clinical Pharmacology states that offending drugs should be discontinued in patients who develop an increase of >60 ms in QTc-interval [7]. Additionally, “Practice Standards for Electrocardiographic Monitoring in Hospital Setting” published by the American Heart Association (AHA) states that offending drugs should be discontinued in patients who develop a prolonged QTc-interval >500 ms [8]. In our study, ten patients demonstrated either a QTc-interval >500 ms, or an increase of >60 ms in QTC-interval on follow-up EKG. Prescribers infrequently responded with therapy modifications, which may have led to one patient experiencing torsades de pointes. To avoid the potential development of fatal arrhythmias in the setting of a prolonged QTc-interval, alternative antibiotics could be utilized. The Infectious Disease Society of America provides alternative recommendations for specific disease states, and an antibiotic that does not prolong the QTc-interval could be selected. Pharmacists infrequently recommended follow-up monitoring or offered alternative treatment recommendations. Lack of documentation may have led to the perceived small amount of pharmacist interventions. Pharmacists were also not able to view follow-up EKGs, as results are not readily reposted in the electronic medical record. Additional limitations to the study include its small sample size, and other medications or patient-specific characteristics that cause QTc-prolongation were not assessed. Our study showed that the QTc-interval was not optimally monitored in some instances for patients, despite recommendations from manufacturers. For instance, six patients did not receive a baseline EKG, and 13 patients did not receive a follow-up EKG. These results may also be experienced in other institutions, stressing the importance of inter-professional communication to place an emphasis on follow-up monitoring or use of alternative therapy agents to avoid the drug–drug interaction altogether.

## Figures and Tables

**Table 1:** Electrocardiogram (EKG) results and subsequent therapy modifications. QTc: corrected QT-interval.

Baseline EKG obtained (%)	36/42 (85.7)
Median male QTc, ms (range)	473 (405–602)
Male QTc >450 ms (%)	9/14 (62.4%)
Median female QTc, ms (range)	470 (435–599)
Female QTc >470 ms (%)	12/22 (54.5%)
Follow-up EKG recommended by pharmacist (%)	3/36 (8.3)
Follow-up EKG obtained (%)	23/36 (63.8)
Median male QTc, ms (range)	481 (440–628)
Male QTc >450 ms (%)	2/8 (25)
Median female QTc, ms (range)	484 (384–645)
Female QTc >470 ms (%)	11/15 (73.3)
Follow-up EKG QTc-interval >500 ms, or QTc-interval increase of >60 ms (%)	10/23 (43.5)
Therapy changed based on follow-up EKG (%)	3/23 (13)
Patient developed torsades de pointes (%)	1/23 (4.3)
Patient QTc-interval increased to >600 ms (%)	2/23 (8.7)
